# Supplementary material for: Chromosome architecture and low cohesion bias acrocentric chromosomes towards aneuploidy during mammalian meiosis
Source: Nat Commun. 2024 Dec 23;15:10713. doi: 10.1038/s41467-024-54659-3 (PMC11666783; doi:10.1038/s41467-024-54659-3)
Supplement: Supplementary file 1 — Supplementary Information [file 41467_2024_54659_MOESM1_ESM.pdf]

Supplementary Information

Chromosome architecture and low cohesion bias acrocentric chromosomes towards aneuploidy during mammalian meiosis

Authors: Eirini Bellou[1], Agata P. Zielinska[1], Eike Urs Mönnich[1], Nina Schweizer[1], Antonio Z. Politi[1], Antonina Wellecke[1], Claus Sibold[2], Andreas Tandler-Schneider[2], Melina Schuh[1*]

Affiliations:

[1] Max Planck Institute for Multidisciplinary Sciences, Göttingen, Germany

[2] Fertility Center Berlin, Berlin, German

[*]Corresponding author. Email: melina.schuh@mpinat.mpg.de

Inventory:

- Supplementary Figure 1-5
- Supplementary Table 1

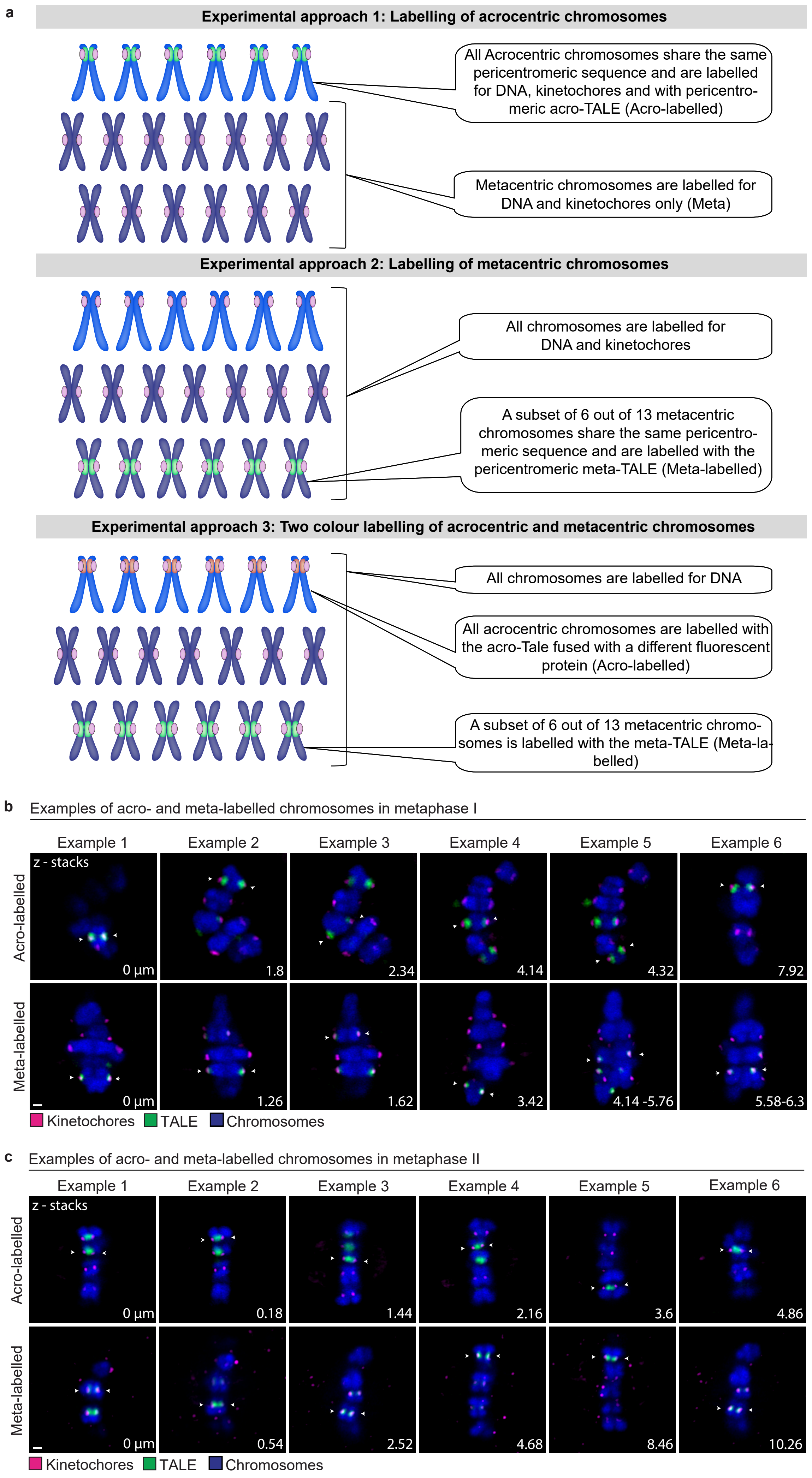

a
Experimental approach 1: Labelling of acrocentric chromosomes
All Acrocentric chromosomes share the same pericentromeric sequence and are labelled for DNA, kinetochores and with pericentromeric acro-TALE (Acro-labelled)
Metacentric chromosomes are labelled for DNA and kinetochores only (Meta)
Experimental approach 2: Labelling of metacentric chromosomes
All chromosomes are labelled for DNA and kinetochores
A subset of 6 out of 13 metacentric chromosomes share the same pericentromeric sequence and are labelled with the pericentromeric meta-TALE (Meta-labelled)
Experimental approach 3: Two colour labelling of acrocentric and metacentric chromosomes
All chromosomes are labelled for DNA
All acrocentric chromosomes are labelled with the acro-Tale fused with a different fluorescent protein (Acro-labelled)
A subset of 6 out of 13 metacentric chromosomes is labelled with the meta-TALE (Meta-labelled)
b
Examples of acro- and meta-labelled chromosomes in metaphase I
Example 1
Example 2
Example 3
Example 4
Example 5
Example 6
z - stacks
Acro-labelled
0 µm
1.8
2.34
4.14
4.32
7.92
Meta-labelled
0 µm
1.26
1.62
3.42
4.14 -5.76
5.58-6.3
Kinetochores
TALE
Chromosomes
c
Examples of acro- and meta-labelled chromosomes in metaphase II
Example 1
Example 2
Example 3
Example 4
Example 5
Example 6
z - stacks
Acro-labelled
0 µm
0.18
1.44
2.16
3.6
4.86
Meta-labelled
0 µm
0.54
2.52
4.68
8.46
10.26
Kinetochores
TALE
Chromosomes

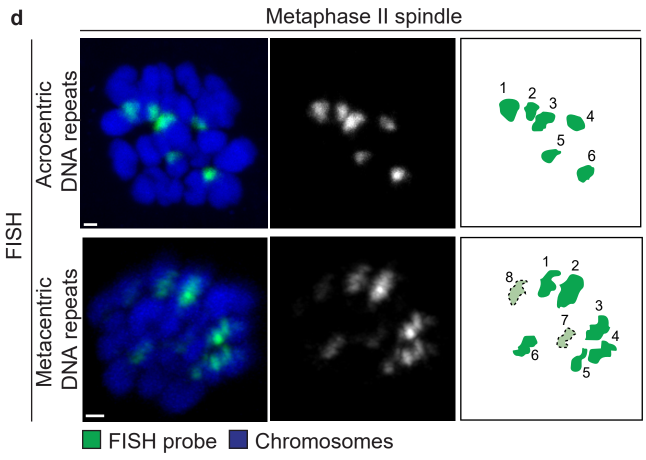
d
Metaphase II spindle
FISH
Acrocentric DNA repeats
Metacentric DNA repeats
1
2
3
4
5
6
1
2
3
4
5
6
7
8
FISH probe
Chromosomes

Supplementary Figure 1. Acrocentric chromosomes missegregate more often than metacentric chromosomes in porcine oocytes.

a, Illustration of the labelling system in the porcine oocytes for the three different experimental approaches. Top panel: Labelling of the acrocentric chromosomes with the acrocentric-TALE. Middle panel: labelling of a subset of the metacentric chromosomes with the metacentric-TALE. 10 of the metacentric chromosomes are expected to share the same pericentromeric sequence with a different number of repeats. Only the six chromosomes with the highest number of repeats are visible under the microscope. Occasionally, in fixed cells a seventh chromosome can be visible with weak signal. Bottom panel: Simultaneous labelling of acrocentric and some of the metacentric chromosomes, using both TALEs. Explanation of what is labelled in each experiment and the respective nomenclature can be found on the right side of the illustration. Magenta, kinetochores; green, acrocentric-TALE (top panel); green, metacentric-TALE (middle and bottom panel); orange, acrocentric-TALE (bottom panel); blue, chromosomes. **b,** Single (z-planes) or a projection of a few z-planes of Airyscan immunofluorescence images of labelled chromosomes with the acrocentric-TALE (top row) and the metacentric TALE (bottom row). Images are from a metaphase I intact porcine oocyte injected with the respective TALE. Magenta, kinetochores, (ACA); green, TALE (anti-GFP); blue, chromosomes, (Hoechst). Scale bar, 1 µm. **c,** Single z-planes of Airyscan immunofluorescence images of labelled chromosomes with the acrocentric TALE (top row) and the metacentric TALE (bottom row). Images of a meiosis II intact porcine eggs injected with the respective TALE. Magenta, kinetochores, (ACA); green, TALE (anti-GFP); blue, chromosomes, (Hoechst). Scale bar, 1 µm. **d,** Representative immunofluorescence images of intact fixed metaphase II eggs with FISH probes targeting the same loci as the acrocentric-TALE (top row) or the metacentric-TALE (bottom row). Numbers next to the green illustrations indicate the number of acro-labelled or meta-labelled chromosomes. The light green spots with the dashed line in the meta-labelled panel are three extra meta-labelled chromosomes with less repeats that are occasionally visible in fixed cells in favorable imaging conditions. Scale bar, 1 µm**.** Representative example of 21 porcine oocytes labelled with FISH probes.

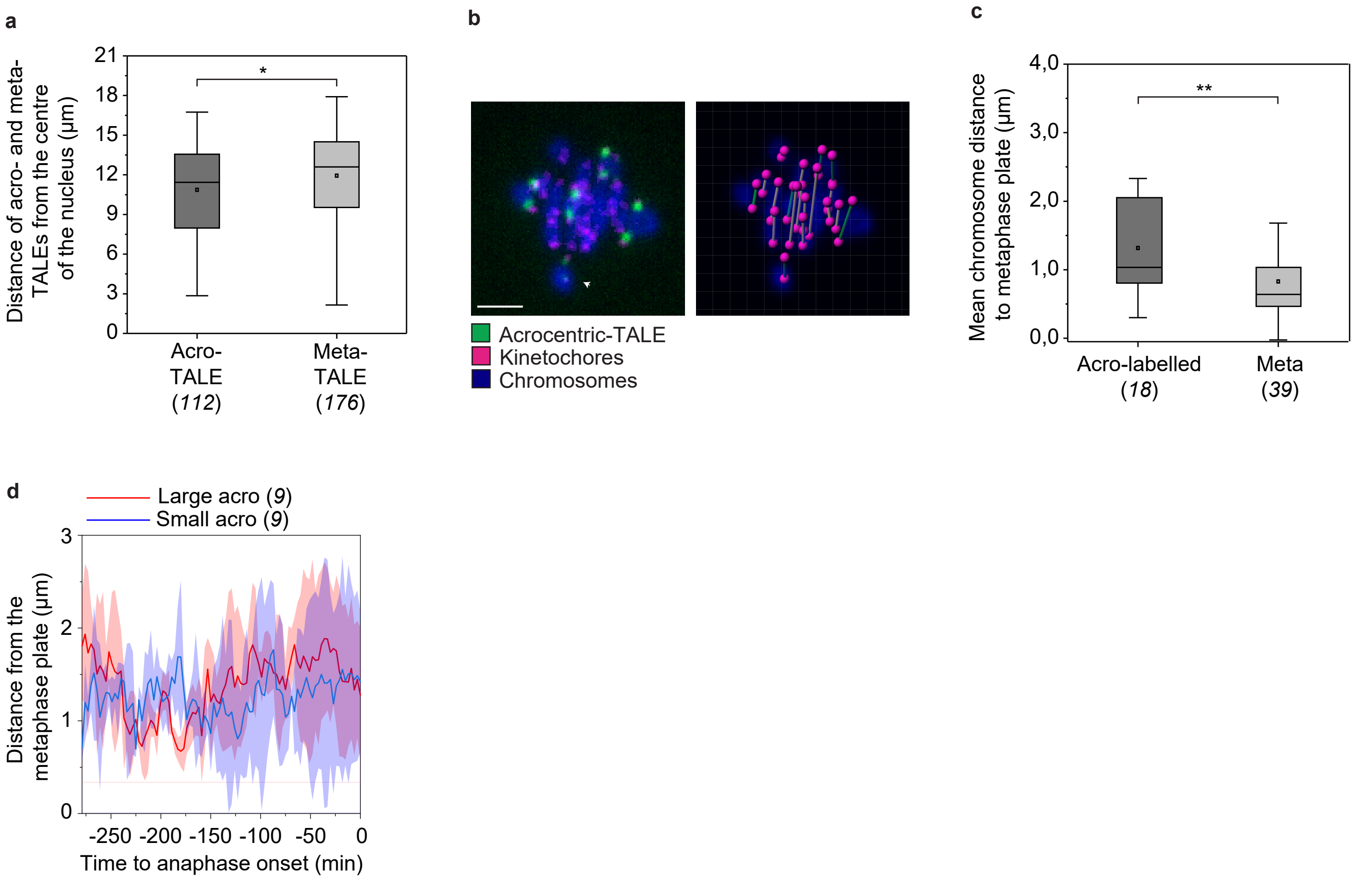

a
Distance of acro- and meta-TALEs from the centre of the nucleus (µm)
*
Acro-TALE (112)
Meta-TALE (176)
b
Acrocentric-TALE
Kinetochores
Chromosomes
c
Mean chromosome distance to metaphase plate (µm)
**
Acro-labelled (18)
Meta (39)
d
Large acro (9)
Small acro (9)
Distance from the metaphase plate (µm)
Time to anaphase onset (min)

Supplementary Figure 2. Acrocentric chromosomes are more likely to misalign during late metaphase I and to lag during anaphase.

a, Distance of acro- and meta-TALE from the centre of the nucleus (22 oocytes analysed). Box plot shows median (horizontal black line), mean (small black square), 25th and 75th percentile (boxes), outliers (whiskers). Two-tailed unpaired t-test (p=0.03518). **b,** Still images from porcine chromosomes during alignment in the metaphase plate (left-hand side) and the corresponding image from Imaris with the pairing annotation (right-hand side). White arrowhead indicates the misaligned acro-labelled chromosome. On the left, magenta, kinetochores (mScarlet-hCENPC); green, acrocentric label, (Acrocentric-TALE-GFP); blue, chromosomes, (H2B-SNAPf) and on the right magenta, kinetochores; green line, pair of homologous kinetochores of acro-labelled chromosomes; white line, pair of homologous kinetochores of meta chromosomes. Scale bar, 1 µm. **c,** Box plot showing the mean distance of acro-labelled and meta chromosomes to the metaphase plate. The mean distance has been calculated from a time series spanning 279 min before polar body extrusion (3 oocytes analysed). Box plot shows median (horizontal black line), mean (small black square), 25th and 75th percentile (boxes), SD (whiskers). Two-tailed unpaired t-test (p=0.00666). **d,** Quantifications of the distance of small and large acro-labelled chromosomes from the centre of the metaphase plate. Error bars (shaded areas) represent SD (3 oocytes analysed).

The number of chromosomes is indicated in brackets under each category. P-values in the graphs are indicated as follows, *p<0.05 and **p< 0.01. The number of cells is indicated in brackets in the figure legend.

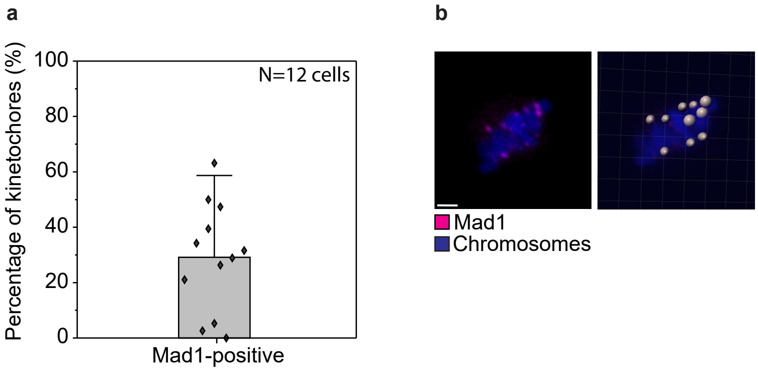

a
b
Percentage of kinetochores (%)
100
80
60
40
20
0
N=12 cells
Mad1-positive
Mad1
Chromosomes

Supplementary Figure 3. Porcine acrocentric chromosomes frequently form erroneous kinetochore-microtubule attachments.

a, Percentage of Mad1-positive kinetochores at late MI (12 h post release), in porcine oocytes (133 Mad1-positive spots from 12 oocytes analysed). **b,** Still images from mad1 positive kinetochores during alignment in the metaphase plate in porcine oocytes (left-hand side) and the corresponding image from Imaris with spots for the Mad1 positive kinetochores (right-hand side). On the left, magenta, Mad1 positive kinetochores (mScarlet-mad1); blue, chromosomes, (H2B-SNAPf) and on the right magenta, kinetochores; blue, chromosomes; white spots, Mad1 positive signal detected. Scale bar, 1 µm.

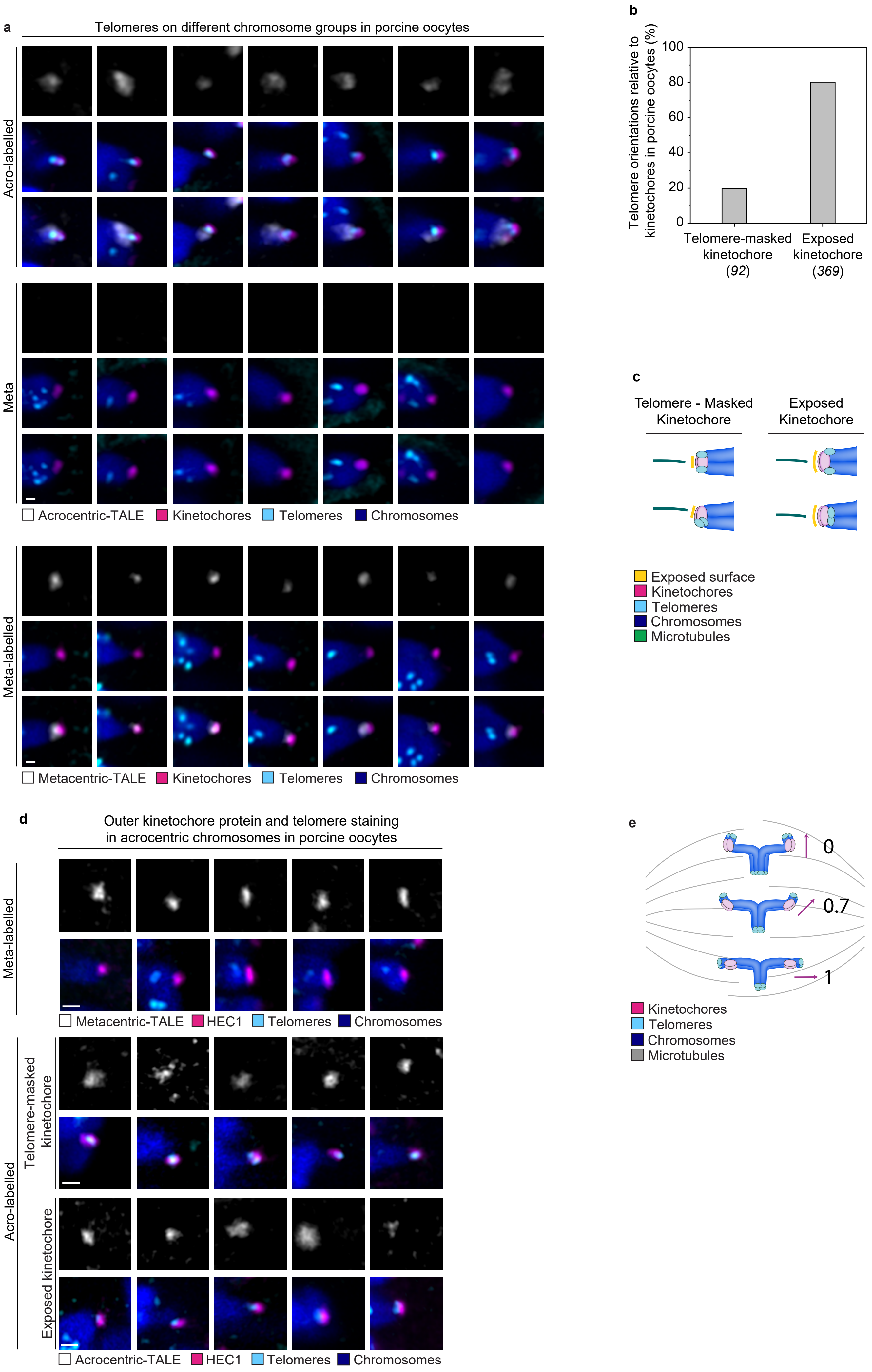
a
Telomeres on different chromosome groups in porcine oocytes
Acro-labelled
Meta
Acrocentric-TALE
Kinetochores
Telomeres
Chromosomes
Meta-labelled
Metacentric-TALE
Kinetochores
Telomeres
Chromosomes
b
Telomere orientations relative to kinetochores in porcine oocytes (%)
100
80
60
40
20
0
Telomere-masked kinetochore (92)
Exposed kinetochore (369)
c
Telomere - Masked Kinetochore
Exposed Kinetochore
Exposed surface
Kinetochores
Telomeres
Chromosomes
Microtubules
d
Outer kinetochore protein and telomere staining in acrocentric chromosomes in porcine oocytes
Meta-labelled
Metacentric-TALE
HEC1
Telomeres
Chromosomes
Acro-labelled
Telomere-masked kinetochore
Exposed kinetochore
Acrocentric-TALE
HEC1
Telomeres
Chromosomes
e
0
0.7
1
Kinetochores
Telomeres
Chromosomes
Microtubules

Supplementary Figure 4. Kinetochores partially-masked by the small chromosome arm are more likely to be incorrectly attached to microtubules.

a, Representative examples of immunofluorescence Airyscan images of telomeres on the different chromosome classes in meiosis I porcine oocytes. Acrocentric chromosomes (Acro-labelled) have telomeres in the proximity of the kinetochores while metacentric chromosomes (meta and meta-labelled) do not have telomeres in the proximity of kinetochores. Magenta, kinetochores, (ACA); cyan, telomeres, (TRF-2); grey, acrocentric-TALE (Acrocentric-TALE-GFP)/metacentric-TALE (Metacentric-TALE-GFP); blue, chromosome, (Hoechst). Scale bar, 0.5 µm **b,** Proportion of telomere-masked and exposed kinetochores in acrocentric chromosomes from the total kinetochores examined. Number of chromosomes analysed is indicated in brackets under each category (44 oocytes analysed). **c,** Illustrations show different possible orientations of telomeres with respect to the kinetochore in telomere-masked and exposed kinetochores. Magenta, kinetochores; cyan, telomeres; blue, chromosomes; green, microtubules; yellow, exposed surface. **d,** Representative examples of immunofluorescence Airyscan images of the outer kinetochore protein Hec1 and telomeres in acrocentric chromosomes in meiosis I porcine oocytes. Magenta, kinetochores, (Hec1); cyan, telomeres, (TRF-2); grey, acrocentric-TALE (Acrocentric-TALE-GFP); blue, chromosome, (Hoechst). Scale bar, 1 µm. **e,** Examples of different possible angles of kinetochores of acrocentric chromosomes. Purple arrows indicate the long axis of the kinetochore, and black arrows indicate the spindle axis. The number indicates the respective dot product. The number of kinetochores analysed is indicated in brackets.

a

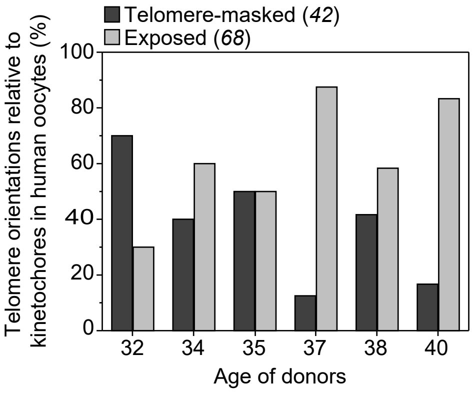
Telomere-masked (*42*)
Exposed (*68*)
Telomere orientations relative to kinetochores in human oocytes (%)
100
80
60
40
20
0
32
34
35
37
38
40
Age of donors

Supplementary Figure 5. Human acrocentric chromosomes frequently form erroneous kinetochore-microtubule attachments.

a, Percentage of telomere-masked and exposed kinetochores in acrocentric chromosomes of human oocytes in relation to the age of the donor (10 human oocytes analysed). Number of kinetochores analysed is indicated in brackets next to each category.

Cell	Kinetochores	Acro-labelled chromosomes	Acro-labelled kinetochores	Meta chromosomes	Meta kinetochores
**Expected in euploid porcine cell**	**38**	**6**	**12**	**13**	**26**
1	38	6	12	13	26
2	34	4	8	13	26
3	38	6	12	13	26
4	38	6	12	13	26
5	38	6	12	13	26
6	38	6	12	13	26
7	38	6	12	13	26
8	36	5	10	13	26
9	38	6	12	13	26
10	38	6	12	13	26
11	38	6	12	13	26
12	38	6	12	13	26
13	38	6	12	13	26
14	38	6	12	13	26
15	38	6	12	13	26
16	34	4	8	13	26
17	38	6	12	13	26
18	42	8	16	13	26
19	38	6	12	13	26
20	34	4	8	13	26
21	38	6	12	13	26
22	38	6	12	13	26
23	38	6	12	13	26
24	39	6	12	13,5	27
25	38	6	12	13	26
26	38	6	12	13	26
27	36	5	10	13	26
28	38	6	12	13	26
29	38	6	12	13	26
30	38	6	12	13	26
31	38	6	12	13	26
32	38	6	12	13	26
33	38	6	12	13	26
34	48	9	18	15	30
35	38	6	12	13	26
36	38	6	12	13	26
37	36	4	8	14	28
38	38	6	12	13	26
39	38	6	12	13	26
40	38	6	12	13	26
41	38	6	12	13	26
42	38	6	12	13	26
43	38	6	12	13	26
44	38	6	12	13	26
45	38	6	12	13	26
46	38	6	12	13	26
47	38	6	12	13	26
48	36	5	10	13	26
49	38	6	12	13	26
50	38	6	12	13	26
51	38	6	12	13	26
52	36	5	10	13	26
53	32	3	6	13	26
54	42	7	14	14	28
55	38	6	12	13	26
56	44	8	16	14	28
57	38	6	12	13	26
58	42	8	16	13	26
59	34	5	10	12	24
60	38	6	12	13	26
61	38	6	12	13	26
62	36	5	10	13	26
63	38	6	12	13	26
64	36	5	10	13	26
65	38	6	12	13	26
66	38	6	12	13	26
67	36	5	10	13	26
68	38	6	12	13	26
69	40	7	14	13	26
70	38	6	12	13	26
71	38	6	12	13	26
72	40	7	14	13	26
73	40	7	14	13	26
74	40	7	14	13	26
75	32	4	8	12	24
76	36	5	10	13	26
77	38	6	12	13	26
78	34	3	6	14	28
79	38	6	12	13	26
80	38	6	12	13	26
81	38	6	12	13	26
82	38	6	12	13	26
83	38	6	12	13	26
84	38	6	12	13	26
85	42	7	14	14	28
86	38	6	12	13	26
87	38	6	12	13	26
88	38	6	12	13	26
89	38	6	12	13	26
90	38	6	12	13	26
91	38	6	12	13	26
92	38	6	12	13	26
93	36	5	10	13	26
94	38	6	12	13	26
95	38	6	12	13	26
96	38	6	12	13	26

Supplementary Table 1. Number of kinetochores for each individual egg in Figure 1e. Table with numbers of all kinetochores, and all acro-labelled chromosomes analysed for every egg used in Figure 1e.
