# Supplementary material for: Chromosome architecture and low cohesion bias acrocentric chromosomes towards aneuploidy during mammalian meiosis
Source: Nat Commun. 2024 Dec 23;15:10713. doi: 10.1038/s41467-024-54659-3 (PMC11666783; doi:10.1038/s41467-024-54659-3)
Supplement: Supplementary file 2 — Description of Additional Supplementary Files [file 41467_2024_54659_MOESM2_ESM.pdf]

Description of Additional Supplementary Files:

Supplementary Movie 1: Porcine oocyte labelled with the acrocentric-TALE. Airyscan sections of a porcine oocyte injected with the acrocentric-TALE and stained for kinetochores, (magenta, ACA); TALE (green, anti-GFP); chromosomes, (blue, Hoechst). Arrowheads indicate the acrocentric chromosomes labelled with the acrocentric –TALE. One section corresponds to 0.18 µm, as indicated.

Supplementary Movie 2: Porcine oocyte labelled with the metacentric-TALE. Airyscan sections of a porcine oocyte injected with the metacentric-TALE and stained for kinetochores, (magenta, ACA); TALE (green, anti-GFP); chromosomes, (blue, Hoechst). Arrowheads indicate the subset of six metacentric chromosomes labelled with the metacentric-TALE. Occasionally in fixed oocytes, a seventh meta-labelled chromosome might be visible (-2.34 µm in the video). One section corresponds to 0.18 µm, as indicated.

Supplementary Movie 3: Anaphase in porcine oocytes without lagging chromosomes. Time-lapse movie of chromosome segregation in porcine oocytes without lagging chromosomes during anaphase I. Time, hours:minutes, 00:00 is anaphase onset. Magenta, kinetochores (mScarlethCENPC); green, acrocentric label, (Acrocentric-TALE-GFP); blue, chromosomes, (H2B-SNAPf). Zprojections 21 sections every 1 µm.

Supplementary Movie 4: Anaphase in porcine oocytes with mildly lagging chromosomes. Time-lapse movie of chromosome segregation in porcine oocytes with mildly lagging chromosome during anaphase I. The lagging chromosome is metacentric as it is not labelled with the acrocentricTALE. Arrowhead indicates the mildly lagging chromosome. Time, hours:minutes, 00:00 is anaphase onset. Magenta, kinetochores (mScarlet-hCENPC); green, acrocentric label, (Acrocentric-TALE-GFP); blue, chromosomes, (H2B-SNAPf). Z-projections 22 sections every 1 µm.

Supplementary Movie 5: Anaphase in porcine oocytes with severely lagging chromosomes. Time-lapse movie of chromosome segregation in porcine oocytes with a severely lagging chromosome during anaphase I, which eventually missegregates. The lagging chromosome is acrocentric as it is labelled with the acrocentric-TALE. Arrowhead indicates the severely lagging chromosome. Time, hours:minutes, 00:00 is anaphase onset. Magenta, kinetochores (mScarlet-hCENPC); green, acrocentric label, (Acrocentric-TALE-GFP); blue, chromosomes, (H2B-SNAPf). Z-projections 24 sections every 1 µm.

Supplementary Movie 6: Rotating 3D metacentric chromosome, example 1. Single chromosome movie with maximum intensity projection.

Supplementary Movie 7: Rotating 3D metacentric chromosome, example 2. Single chromosome movie with maximum intensity projection.

Supplementary Movie 8: Rotating 3D metacentric chromosome, example 3. Single chromosome movie with maximum intensity projection.

Supplementary Movie 9: Rotating 3D acrocentric chromosome with telomere-masked kinetochore, example 1. Single chromosome movie with maximum intensity projection.

Supplementary Movie 10: Rotating 3D acrocentric chromosome with telomere-masked kinetochore, example 2. Single chromosome movie with maximum intensity projection.

Supplementary Movie 11: Rotating 3D acrocentric chromosome with telomere-masked kinetochore, example 3. Single chromosome movie with maximum intensity projection.

Supplementary Movie 12: Rotating 3D acrocentric chromosome with exposed kinetochore, example 1. Single chromosome movie with maximum intensity projection.

Supplementary Movie 13: Rotating 3D acrocentric chromosome with exposed kinetochore, example 2. Single chromosome movie with maximum intensity projection.

Supplementary Movie 14: Rotating 3D acrocentric chromosome with exposed kinetochore, example 3. Single chromosome movie with maximum intensity projection.
