# Supplementary material for: Chromosome architecture and low cohesion bias acrocentric chromosomes towards aneuploidy during mammalian meiosis
Source: Nat Commun. 2024 Dec 23;15:10713. doi: 10.1038/s41467-024-54659-3 (PMC11666783; doi:10.1038/s41467-024-54659-3)
Supplement: Supplementary file 17 — Reporting Summary [file 41467_2024_54659_MOESM17_ESM.pdf]

nature portfolio

Corresponding author(s): Melina Schuh

Last updated by author(s): Nov 9, 2024

# Reporting Summary

Nature Portfolio wishes to improve the reproducibility of the work that we publish. This form provides structure for consistency and transparency in reporting. For further information on Nature Portfolio policies, see our Editorial Policies and the Editorial Policy Checklist.

## Statistics

For all statistical analyses, confirm that the following items are present in the figure legend, table legend, main text, or Methods section.

| n/a | Confirmed | |
|---|---|---|
| ☐ | ☒ | The exact sample size (*n*) for each experimental group/condition, given as a discrete number and unit of measurement |
| ☐ | ☒ | A statement on whether measurements were taken from distinct samples or whether the same sample was measured repeatedly |
| ☐ | ☒ | The statistical test(s) used AND whether they are one- or two-sided<br>*Only common tests should be described solely by name; describe more complex techniques in the Methods section.* |
| ☒ | ☐ | A description of all covariates tested |
| ☐ | ☒ | A description of any assumptions or corrections, such as tests of normality and adjustment for multiple comparisons |
| ☐ | ☒ | A full description of the statistical parameters including central tendency (e.g. means) or other basic estimates (e.g. regression coefficient) AND variation (e.g. standard deviation) or associated estimates of uncertainty (e.g. confidence intervals) |
| ☐ | ☒ | For null hypothesis testing, the test statistic (e.g. *F*, *t*, *r*) with confidence intervals, effect sizes, degrees of freedom and *P* value noted<br>*Give P values as exact values whenever suitable.* |
| ☒ | ☐ | For Bayesian analysis, information on the choice of priors and Markov chain Monte Carlo settings |
| ☒ | ☐ | For hierarchical and complex designs, identification of the appropriate level for tests and full reporting of outcomes |
| ☒ | ☐ | Estimates of effect sizes (e.g. Cohen's *d*, Pearson's *r*), indicating how they were calculated |

*Our web collection on statistics for biologists contains articles on many of the points above.*

## Software and code

Policy information about availability of computer code

| | |
|---|---|
| Data collection | Zeiss Zen software (Version 2.3) or Zen Black 2.1 were used to acquire the confocal images. Microscope 1.0.5.2 software was used in order to acquire the light-sheet data. Imspector software was used for acquiring the STED data. |
| Data analysis | Images were processed using Zeiss Zen software (Version 2.3 blue edition) or Zen Black respectively, and analysed with Bitplane Imaris (Version 9.3 and/or Fiji (Fiji Is Just ImageJ) and/or Arivis Vision4D (version 3.1.1) as described in the Methods section. In-house scripts for analysis with the above software packages were written in Matlab R2018b, ImageJ or R and are available under the following link: https://doi.org/10.17617/3.NPIDK2 |

For manuscripts utilizing custom algorithms or software that are central to the research but not yet described in published literature, software must be made available to editors and reviewers. We strongly encourage code deposition in a community repository (e.g. GitHub). See the Nature Portfolio guidelines for submitting code & software for further information.

## Data

Policy information about availability of data

All manuscripts must include a data availability statement. This statement should provide the following information, where applicable:

- Accession codes, unique identifiers, or web links for publicly available datasets
- A description of any restrictions on data availability
- For clinical datasets or third party data, please ensure that the statement adheres to our policy

Source data are provided with this paper. The datasets generated and analysed as part of the current study are available from the corresponding author on request.

Due to their large size, the primary microscopy data were not uploaded to a data repository but are available from the corresponding author on request.

## Research involving human participants, their data, or biological material

Policy information about studies with human participants or human data. See also policy information about sex, gender (identity/presentation), and sexual orientation and race, ethnicity and racism.

| | |
|---|---|
| Reporting on sex and gender | In this study all donors were of female sex, as oocytes can only be collected from female donors. |
| Reporting on race, ethnicity, or other socially relevant groupings | All donations were provided anonymously. |
| Population characteristics | All donations were provided anonymously. |
| Recruitment | All human oocytes were collected from patients at Fertility Center Berlin. Oocytes were collected from patients who underwent ovarian stimulation for intracytoplasmic sperm injection (ICSI) as part of their assisted reproduction treatment at Fertility Center Berlin. Only oocytes that were immature at the time of ICSI and thus unsuitable for the procedure were vitrified for this study. All patients gave informed consent for their surplus oocyte(s) to be used in this study. |
| Ethics oversight | The use of unfertilized human oocytes in this study was approved by the Ärztekammer Niedersachsen (Ethics Committee of Lower Saxony) under the reference 15/2016. |

Note that full information on the approval of the study protocol must also be provided in the manuscript.

# Field-specific reporting

Please select the one below that is the best fit for your research. If you are not sure, read the appropriate sections before making your selection.

[x] Life sciences   [ ] Behavioural & social sciences   [ ] Ecological, evolutionary & environmental sciences

For a reference copy of the document with all sections, see nature.com/documents/nr-reporting-summary-flat.pdf

# Life sciences study design

All studies must disclose on these points even when the disclosure is negative.

| | |
|---|---|
| Sample size | No statistical methods were used to predetermine sample size. Sample size was determined by the number of oocytes available every time. For each replicate the oocytes were collected from multiple porcine ovaries that arrived from the slaughterhouse on the respective day. Oocytes from different animals were pooled together and the total number was then separated into the experimental groups. Attention was paid that cells were distributed similarly between the experimental groups. Further parameters like survival rate of oocytes upon injections affected the final sample size. |
| Data exclusions | Data were excluded from analyses when imaging was unsuccessful. Due to the large size of porcine oocytes and their opaque nature when the spindle was positioned away from the coverslip (within third of the cell that was located away from the coverslip), the cells could not be imaged and were hence excluded from the analysis. Cells that died during imaging were excluded from the analysis. For some experiments, the analysis was only possible for spindles that were parallel to the imaging plane. Thus, any spindles with a non-parallel orientation were excluded from the analysis. |
| Replication | All data are from at least three independent experiments apart from the cold stable assay in metaphase II of porcine oocytes and the FISH labelling which are from two independent repetitions. All attempts at replication were successful. |
| Randomization | Samples were collected from multiple animals each time. All cells were pooled together and then randomly split into the experimental groups to ensure equivalence between groups. |
| Blinding | The analysis of kinetochore-microtubule attachments was conducted blindly. First, the attachments were categorised into the respective groups without knowing if the kinetochore belongs to an acrocentric or metacentric chromosome. After finishing the assessment of all kinetochores, the imaging channel of the TALE was turned on and the kinetochores were assigned to acrocentric and metacentric chromosomes. The other experimental outcomes were not analysed blindly, but automated software was used for analyses wherever possible. |

# Reporting for specific materials, systems and methods

We require information from authors about some types of materials, experimental systems and methods used in many studies. Here, indicate whether each material, system or method listed is relevant to your study. If you are not sure if a list item applies to your research, read the appropriate section before selecting a response.

## Materials & experimental systems

| n/a | Involved in the study |
| --- | --- |
| ☐ | ☒ Antibodies |
| ☒ | ☐ Eukaryotic cell lines |
| ☒ | ☐ Palaeontology and archaeology |
| ☐ | ☒ Animals and other organisms |
| ☒ | ☐ Clinical data |
| ☒ | ☐ Dual use research of concern |
| ☒ | ☐ Plants |

## Methods

| n/a | Involved in the study |
| --- | --- |
| ☒ | ☐ ChIP-seq |
| ☒ | ☐ Flow cytometry |
| ☒ | ☐ MRI-based neuroimaging |

# Antibodies

Antibodies used

Primary antibodies used were human anti-centromere antibody (ACA) (Antibodies Incorporated #15-234), rabbit anti-GFP (#A11122, Invitrogen), rat anti-alpha-tubulin (MCA78G, Bio-rad), mouse anti-TRF-2 (NB100-56506SS, Novus Biologicals), goat anti-GFP (600-101-215; Rockland Immunochemicals), mouse anti-alpha-tubulin (#T6199, Merck), rabbit anti-pH3 (#9701, Cell Signaling Technology), rabbit anti-REC8 generated in-house based on a published epitope (Eijpe, M., J Cell Biol, 2003), rabbit anti-SMC3 (#ab128919, abcam), mouse anti-HEC1 (#ab3613,Abcam).

Secondary antibodies used in this study were: Goat anti-Human IgG (H+L) Cross-Adsorbed Secondary Antibody, Alexa Fluor 647 (#A-21445, ThermoFisher), Goat anti-Human IgG (H+L) Cross-Adsorbed Secondary Antibody, Alexa Fluor 488 (#A-11013, ThermoFisher), Donkey anti-Rabbit IgG (H+L) Highly Cross-Adsorbed Secondary Antibody, Alexa Fluor 488 (#A-21206, ThermoFisher), Goat anti-Rabbit IgG (H+L) Highly Cross-Adsorbed Secondary Antibody, Alexa Fluor 568 (#A-11036, ThermoFisher), Donkey anti-Mouse IgG (H+L) Highly Cross-Adsorbed Secondary Antibody, Alexa Fluor 568 (#A-10037, ThermoFisher), Donkey anti-Mouse IgG (H +L) Highly Cross-Adsorbed Secondary Antibody, Alexa Fluor488 (#A-21202, ThermoFisher), Goat anti-Rat IgG (H+L) Cross-Adsorbed Secondary Antibody, Alexa Fluor 488 (#A-11006, ThermoFisher), Goat anti-Rat IgG (H+L) Cross-Adsorbed Secondary Antibody, Alexa Fluor 568 (#A-11077, ThermoFisher), Donkey anti-Goat IgG (H+L) Cross-Adsorbed Secondary Antibody, Alexa Fluor 568 (#A-11057, ThermoFisher), Donkey Anti-Rabbit IgG H&L Alexa Fluor 405 preadsorbed (#ab175649, Abcam), Rhodamine Red-X (RRX) AffiniPure Fab Fragment Goat Anti-Mouse IgG2a, Fcγ fragment specific (#115-297-186, Jackson ImmunoResearch), Alexa Fluor 647 AffiniPure Fab Fragment Goat Anti-Mouse IgG1, Fcγ fragment specific (#115-607-185, Jackson ImmunoResearch), Donkey anti human STAR RED (#STRED-1054-500UG, Abberior Gmbh), Goat anti rabbit STAR ORANGE (#STORANGE-1002-500UG, Abberior Gmbh).

Validation

The human anti-centromere antibody -ACA, Antibodies Incorporated #15-234, is tested for by the manufacturer for specificity to hamster, human, mouse and rat cells. In this paper we validated the antibody with immunofluorescence in porcine oocytes, where it localizes in the centromeres as expected.
The rabbit anti-GFP (#A11122, Invitrogen) is validated by the manufacturer with immunofluorescence and on western blot.
The rat anti-alpha-tubulin (MCA78G, Bio-rad) has verified by the manufacturer on human tubulin and based on sequence similarity it is expected to work in all mammals (statement from the manufacturers webpage). Has been used in 70 publications. We also validated in this paper with immunofluorescence that it localises to spindle microtubules in porcine oocytes.
The mouse anti-TRF-2 (NB100-56506SS) is tested by the manufacturer for specificity on human, mouse, rat, marsupial and Muntjac. the manufacturer states that it can be used in WB, IHC-P, Elisa, flow, IF. In this paper, we validate it with immunofluorescence in porcine oocytes.
The goat anti-GFP (600-101-215; Rockland Immunochemicals), is validated by the manufacturer. The manufacturer has tested it by IF, ELISA, and WB.
The mouse anti-alpha-tubulin (#T6199, Merck) is verified by the manufacturer. It has been tested on yeast, mouse, amphibian, human, rat, chicken, fungi, and bovine. We also validated in this paper with immunofluorescence that it localises to spindle microtubules in porcine oocytes.
The rabbit anti-pH3 (#9701, Cell Signaling Technology), is validated by the manufacturer to work on human, mouse, rat, monkey, and D: melanogaster. The manufacturer states that it can be used in WB, IHC, and IF.
The abbit anti-REC8 generated in-house based on a published epitope (Eijpe, M., J Cell Biol, 2003). In this paper we validated the antibody with immunofluorescence in porcine oocytes, where it localises on the chromosomes arm during meiosis I as expected.
The rabbit anti-SMC3 (#ab128919, abcam), is validated by the manufacturer to react with human. Manufacturers´website states that it is suitable for IHP-C and WB. In this study we validated the antibody with immunofluorescence in porcine eggs, and it localises as expected in the pericentromeric area.
The mouse anti-HEC1 (#ab3613,Abcam) is validated by the manufacturer to react with human and pig samples. The manufacturer states that it is suitable for IP, Flow cyt, WB, and IF. In this study we also validated the antibody by IF in porcine oocytes and it localises as expected in the outer layers of the kinetochore.

# Animals and other research organisms

Policy information about studies involving animals; ARRIVE guidelines recommended for reporting animal research, and Sex and Gender in Research

Laboratory animals

The study did not involve laboratory animals.

Wild animals

The study did not involve wild animals.

| | |
|---|---|
| Reporting on sex | Oocytes can only be collected from female. We only used ovaries from female pigs or immature oocytes from women. |
| Field-collected samples | The study did not involve samples collected from the field. |
| Ethics oversight | No ethical approval or guidance was required for the use of porcine oocytes. The porcine ovaries were collected from a local abattoir as a waste product of the slaughtering process. In Germany where the study was conducted this does not require ethics approval. The use of unfertilized human oocytes in this study was approved by the Ärztekammer Niedersachsen (Ethics Committee of Lower Saxony) under the reference 15/2016. |

Note that full information on the approval of the study protocol must also be provided in the manuscript.

## Plants

| | |
|---|---|
| Seed stocks | No plants were used in this study. |
| Novel plant genotypes | No plants were used in this study. |
| Authentication | No plants were used in this study. |
